# *PARP-1* Variant Rs1136410 Confers Protection against Coronary Artery Disease in a Chinese Han Population: A Two-Stage Case-Control Study Involving 5643 Subjects

**DOI:** 10.3389/fphys.2017.00916

**Published:** 2017-11-14

**Authors:** Xue-bin Wang, Ning-hua Cui, Shuai Zhang, Shu-ren Guo, Ze-jin Liu, Liang Ming

**Affiliations:** ^1^Department of Clinical Laboratory, The First Affiliated Hospital of Zhengzhou University, Zhengzhou, China; ^2^Department of Clinical Laboratory, Children's Hospital of Zhengzhou, Zhengzhou, China; ^3^Center for Gene Diagnosis, Zhongnan Hospital of Wuhan University, Wuhan, China; ^4^Center of Clinical Laboratory, Wuhan Asia Heart Hospital, Wuhan, China

**Keywords:** *PARP-1* rs1136410, PARP activities, coronary artery disease, severity of coronary atherosclerosis, gene-environment interactions

## Abstract

Inhibition of poly(ADP-ribose) polymerase (PARP) may protect against coronary artery disease (CAD) in animal models, and rs1136410, a non-synonymous single nucleotide polymorphism (SNP) in *PARP-1*, has a potential impact on PARP activities *in vitro*. This two-stage case-control study, involving 2803 CAD patients and 2840 controls, aimed to investigate the associations of *PARP-1* rs1136410 with CAD development, lipid levels, PARP activities, 8-hydroxy-2′-dexyguanosine (8-OHdG), and interleukin (IL)-6 levels in a Chinese Han population. Assuming a recessive model, the variant genotype GG of SNP rs1136410 showed a significantly inverse association with CAD risk (adjusted odds ratio (OR) = 0.73, *P* < 0.001), left main coronary artery (LMCA) lesions (*P* = 0.003), vessel scores (*P* = 0.003), and modified Gensini scores (*P* < 0.001). There were significant correlations of SNP rs1136410 with higher levels of total cholesterol (TC) and lower levels of high-density lipoprotein cholesterol (HDL-c). In gene-environment interaction analyses, participants with the variant genotype GG, but without smoking habit, type 2 diabetes mellitus, and hyperlipidemia, conferred an 84% (*P* < 0.001) decreased risk of CAD. The genotype-phenotype correlation analyses further supported the functional roles of SNP rs1136410 in decreasing PARP activities and 8-OHdG levels. Taken together, our data suggest that SNP rs1136410 may confer protection against CAD through modulation of PARP activities and gene-environment interactions in a Chinese Han population.

## Introduction

Coronary artery disease (CAD), the major contributor to death and disability worldwide (Mozaffarian et al., 2016), is a multifactorial disease associated with predisposing genes, metabolic risk factors, and their interactions (Lanktree and Hegele, 2009). Atherosclerosis, the main pathogenesis of CAD, is an oxidative inflammatory process leading to cumulative deposition of lipoproteins, focal intimal thickening, and ultimately myocardial infarction (MI) (He and Zuo, 2015). Oxidative stress, induced by extensive generation of reactive oxygen species (ROS), is a fundamental step of atherosclerosis (Harrison et al., 2003), which triggers multiple DNA lesions of coronary endothelial cells, including modified bases, single- and double- strand breaks (SSB and DSB), and chromosomal aberrations (Shah and Mahmoudi, 2015). In response to these DNA lesions, poly(ADP-ribose) polymerase 1 (PARP-1), a DNA repair sensor, is activated to initiate the base excision repair (BER) pathway by modifying PARP-1 itself and recruiting downstream BER enzymes (El-Khamisy et al., 2003; Altmeyer et al., 2009). However, hyperactivation of PARP-1 by uncontrolled DNA damage consumes excessive NAD^+^ and prevents ATP production, thus causing a cellular energy crisis and cell death (Berger et al., 1983; Xu et al., 2014).

A growing body of evidence pinpointed the critical roles of PARP-1 in the development of CAD. First, in clinical studies, PARP-1 overexpression was observed in human atherosclerotic plaques (Martinet et al., 2002), and increased the circulating levels of proinflammatory cytokines in patients with unstable angina (Huang et al., 2008). Second, catalytic inhibition of PARP selectively promoted sensitization of foam cells to oxidative damage while protecting against oxidant-induced cell death in endothelial and smooth muscle cell (SMC) lines (Hans et al., 2008). Finally, *PARP-1* deletion in mice could ameliorate lipid profiles and endothelium-dependent relaxation (Hans et al., 2009a), reduce expression of proinflammatory factors (von Lukowicz et al., 2008), and consequently block atherosclerotic plaque regression (von Lukowicz et al., 2008; Hans et al., 2009a).

The *PARP-1* gene, located on chromosome 1q41-42, has a well-characterized common single nucleotide polymorphism (SNP), Val762Ala (rs1136410), which is a non-synonymous A-to-G mutation at codon 762, resulting in the conversion of valine to alanine in the catalytic domain of PARP-1 (Yu et al., 2012). *In vitro*, SNP rs1136410 has been reported to exert a dose-dependent impact on kinetics and poly (ADP-ribosyl)ation of PARP (Wang et al., 2007; Beneke et al., 2010).

Taken together, we speculated that SNP rs1136410 might influence PARP activities, and further modulate oxidative DNA damage and CAD risk. Hence, we performed this two-stage case-control study to test whether SNP rs1136410 was associated with CAD risk, CAD severity, lipid levels, 8-hydroxy-2′-dexyguanosine (8-OHdG), interleukin (IL)-6, and PARP activities in a Chinese Han population, followed by multifactor dimensionality reduction (MDR) and classification and regression tree (CART) analyses to identify the high-order gene-environment interactions between SNP rs1136410 and traditional CAD risk factors.

## Materials and methods

### Study subjects

This two-stage case-control study, involving 2803 CAD patients and 2840 age- and sex-matched controls, contained two data sets: the discovery set including 1266 cases and 1296 controls from Wuhan Asia Heart Hospital between March 2011 and February 2017, and the replication set with 1537 cases and 1544 controls from Zhongnan Hospital of Wuhan University and The First Affiliated Hospital of Zhengzhou University (between July 2013 and August 2017). The diagnosis of CAD was based on ≥50% of lumen obstruction in at least one major coronary artery or their main branches by coronary angiography. CAD severity was evaluated by the presence of left main coronary artery (LMCA) lesions, vessel scores, and modified Gensini scores (Montorsi et al., 2006; Weintraub et al., 2011; Bing et al., 2015; Wang et al., 2016; Supplementary Materials and Methods). CAD patients were divided into three groups according to the following clinical presentation: (1) stable angina pectoris (SAP); (2) unstable angina pectoris (UAP); (3) MI (Wang et al., 2016; Supplementary Materials and Methods). The controls were subjects without atherosclerotic lesions and history of CAD, as confirmed by angiography, electrocardiographic test, and physical examination. We collected the following demographics for each participant: (1) traditional CAD risk factors including smoking habit, alcohol drinking habit, and histories of hyperlipidemia, type 2 diabetes mellitus (T2DM), and hypertension; (2) clinical data such as lipid levels, fasting plasma glucose (FPG) levels, blood pressure, and body mass index (BMI) (Supplementary Materials and Methods). We excluded subjects with the following diseases: (1) cardiac diseases such as valvular or congenital heart diseases, myocardial bridge, and coronary spasm; (2) systemic diseases including cancers, hepatic or renal diseases, and autoimmune diseases. This study complied with the Declaration of Helsinki, and was approved by local Ethics Committees. All subjects signed written informed consents accordingly.

### Genotyping for SNP Rs1136410

Genomic DNA of peripheral blood leukocytes was extracted by a phenol-chloroform method. Genotyping for SNP rs1136410 was performed on a LightScanner 96 system (Idaho Technology, Salt Lake City, UT, USA) using high-resolution melting (HRM) analyses (Figure S1; Li et al., 2014). PCR amplification for HRM was conducted with an annealing temperature of 56°C, in 10 μL of PCR mixtures containing 1.5 mM of Mg^2+^, 200 μM of each dNTP, 0.5 μM of each primer (forward primer: 5′-GGG GGC TTT CTT TTG CTC-3′; reverse primer: 5′-TGT CCA GCA GGT TGT CAAG-3′), 1 U of Taq DNA polymerase, 1 μL of LC Green, and 50 ng of genomic DNA. Genotyping results of HRM were verified by direct sequencing and repeated assays in 10% of the whole samples.

### Measurement of PARP activities, 8-OHdG levels, and IL-6 levels in peripheral blood mononuclear cells (PBMCs)

In PBMCs, PARP activities, combined with two well-known biomarkers for oxidative inflammatory response, 8-OHdG and IL-6, were measured with competitive ELISA kits, as described in Supplementary Materials and Methods (Cui et al., 2017). CV values for inter- and intra- assays were 6.4 and 5.5% for PARP activities, 7.2 and 5.1% for 8-OHdG levels, and 4.5 and 6.7% for IL-6 levels, respectively.

### High-order gene-environment interactions between SNP Rs1136410 and traditional CAD risk factors

To evaluate the high-order gene-environment interactions between SNP rs1136410 and traditional CAD risk factors, MDR and CART analyses were conducted by MDR 2.0 (UPenn, Philadelphia, PA, USA) and Clementine 12.0 (SPSS Inc., Chicago, IL, USA) programs, respectively. Briefly, in MDR analyses (Velez et al., 2007), the predictive accuracy for all combinations of included variables was analyzed by 1,000-time permutation and 100-time cross-validation tests. The best interaction model in predicting CAD risk was identified with the maximum cross-validation consistency (CVC) and the optimal testing accuracy.

According to the relative significance of included variables, CART analyses hierarchically subdivided data to produce a binary recursive-partitioning tree, which identified the best interaction model to predict CAD risk using logistic regression analyses (Barnholtz-Sloan et al., 2011). In CART analyses, each split was based on the Gini index, and required a minimal node size of 50 (Gu et al., 2006; Barnholtz-Sloan et al., 2011).

### Statistical analyses

The differences in demographics between cases and controls were compared by the Student's *t*-test (for quantitative variables) and the Pearson χ^2^ test (for qualitative variables). Hardy-Weinberg equilibrium (HWE) for SNP rs1136410 was evaluated by the Pearson χ^2^ test. Allelic and genotypic (additive, recessive, dominant) associations of SNP rs1136410 with CAD risk were tested by logistic regression analyses before and after adjusting for age, sex, smoking status, alcohol drinking status, BMI, and histories of hyperlipidemia, T2DM, and hypertension. The 100,000-time Monte-Carlo permutation test was used to control for multiple testing (Jiang et al., 2014). The homogeneity of ORs between two sets was analyzed by the Breslow-Day test. In subgroup analyses, we used the multiplicative likelihood ratio test to examine the potential gene-environment interactions in CAD risk. The effect of SNP rs1136410 on CAD severity was appraised by the linear-by-linear association χ^2^ test. Multivariable linear regression analyses were conducted to test the associations of SNP rs1136410 with lipid levels, IL-6 levels, 8-OHdG levels, and PARP activities, as well as the effects of IL-6 levels, 8-OHdG levels, or PARP activities on CAD risk and vessel scores. We used the Pearson (for normal distributed data) or Spearman (for skewed data) correlation test to assess the correlations between IL-6 levels, 8-OHdG levels, PARP activities, and modified Gensini scores. All these analyses set *P* < 0.05 (two-tailed) as a significant level in SPSS 17.0 (SPSS Inc., Chicago, IL, USA) and PLINK programs. The statistical power was estimated by PS 3.0 software (Vanderbilt University, Nashville, TN, USA).

## Results

### Population demographics

In both discovery and replication sets (Table S1), CAD patients had higher frequencies of smoking habit, alcohol drinking habit, hyperlipidemia, T2DM, and hypertension, higher levels of BMI, blood pressure, FPG, triglyceride (TG), total cholesterol (TC), and low-density lipoprotein cholesterol (LDL-c), and lower levels of high-density lipoprotein cholesterol (HDL-c) than controls. The genotype distributions of SNP rs1136410 fulfilled expectations of HWE in merged controls (P_HWE_ = 0.228).

### Allelic and genotypic associations between SNP Rs1136410 and CAD risk

In the discovery set, the minor allele G of SNP rs1136410 conferred an 11% reduced risk of CAD [odds ratio (OR) = 0.89, *P* = 0.040, Table 1]. This allelic association was successfully verified in the replication set, with an OR of 0.87 and a *P*-value of 0.006 (Table 1). Since the allelic ORs between discovery and replication sets were homogenous (*P* = 0.733), we performed a meta-analysis of these two sets, which identified a more significant allelic association between SNP rs1136410 and CAD risk, with an OR of 0.88 and a *P*-value of 6.29 × 10^−4^ (Table 1).

**Table 1:** Allelic and genotypic associations of SNP rs1136410 with CAD risk.

**Model**	**Alleles (A/G)/Genotypes**^d^		**Without adjustment**			**With adjustment**^b^	
	**Cases, N**	**Controls, N**	**OR (95%CI)**	**P**	**P_emp_^a^**	**OR (95%CI)**	**P_adj_**
**ALLELIC ASSOCIATION ANALYSES**^c^							
Discovery set	1500/1032	1462/1130	0.89 (0.80−0.99)	0.040	0.031	0.88 (0.78−0.99)	0.034
Replication set	1897/1177	1800/1288	0.87 (0.78−0.96)	0.006	0.005	0.89 (0.80−0.99)	0.032
Merged set	3397/2209	3262/2418	0.88 (0.81−0.95)	6.29 × 10^−4^	6.64 × 10^−4^	0.89 (0.82−0.96)	0.003
**GENOTYPIC ASSOCIATION ANALYSES**^d^							
**Discovery set**							
Additive	423/654/189	411/640/245	0.89 (0.79−0.99)	0.036	0.038	0.88 (0.78−0.99)	0.031
Recessive	1077/189	1051/245	0.75 (0.61−0.93)	0.007	0.008	0.71 (0.57−0.88)	0.002
Dominant	423/843	411/885	0.93 (0.79−1.09)	0.359	0.533	0.94 (0.79−1.12)	0.473
**Replication set**							
Additive	555/787/195	510/780/254	0.86 (0.77−0.96)	0.005	0.009	0.88 (0.79−0.99)	0.027
Recessive	1342/195	1290/254	0.74 (0.60−0.90)	0.003	0.004	0.76 (0.61−0.93)	0.009
Dominant	555/982	510/1034	0.87 (0.75−1.01)	0.073	0.101	0.91 (0.78−1.06)	0.231
**Merged set**							
Additive	978/1441/384	921/1420/499	0.87 (0.81−0.94)	4.62 × 10^−4^	2.31 × 10^−4^	0.88 (0.81−0.96)	0.002
Recessive	2419/384	2341/499	0.75 (0.64−0.86)	6.53 × 10^−5^	8.32 × 10^−5^	0.73 (0.63−0.85)	6.45 × 10^−5^
Dominant	978/1825	921/1919	0.90 (0.80−1.00)	0.051	0.058	0.92 (0.82−1.04)	0.180

a *Emprical P values were obtained from the 100,000-time Monte-Carlo permutation test*.

b *Adjusted OR (95%CI) and P_adj_ values were obtained from logistic regression analyses after adjusting for age, sex, smoking status, alcohol drinking status, BMI, and histories of hyperlipidemia, T2DM, and hypertension*.

c *In allelic association analyses, the major allele A was considered as the reference*.

d *In genotypic association analyses, additive model = AA/AG/GG; recessive model = AG + AA (Reference)/GG.: dominant model = AA (Reference)/GG + AG*.

*N, number; OR (95%CI), odds ratio: (95% confidence interval)*.

In both discovery and replication sets, genotypic association analyses consistently found significant effects of SNP rs1136410 on CAD risk under both additive and recessive models (Table 1). In a meta-analysis of these two sets, the recessive model was identified as the best-fitting model, with the smallest OR of 0.75 and the most significant *P*-value of 6.53 × 10–5 (Table 1). Assuming a recessive OR of 0.75 and a type I error of 0.05, the entire population could offer a statistical power of 97.5% to address the association.

In subtype analyses based on a recessive model (Table S2), the variant genotype GG of SNP rs1136410 was significantly associated with the decreased risk of UAP and MI, with ORs of 0.71 (*P* = 0.002) and 0.61 (*P* = 6.07 × 10–6), respectively. No significant association was found between SNP rs1136410 and SAP.

All significant results remained unchanged after the permutation test for multiple testing and adjustment for covariates (Table 1 and Table S2).

### Gene-environment interactions between SNP Rs1136410 and traditional CAD risk factors

We first used subgroup analyses to seek two-way gene-environment interactions in CAD risk. Assuming a recessive model (Table 2), the variant genotype GG of SNP rs1136410 constantly conferred a reduced risk of CAD in all subgroups, except for subjects with smoking habit, T2DM, and hyperlipidemia. The multiplicative likelihood ratio test further found the two-way interactions of SNP rs1136410 with smoking status (*P*_inter_ = 0.031), history of T2DM (*P*_inter_ = 0.025), and history of hyperlipidemia (*P*_inter_ = 0.010) in decreasing CAD risk.

**Table 2:** Subgroup analyses for the association between SNP rs1136410 and CAD risk.

**Variables**	**SNP rs1136410 (CAD/controls, N)**		**OR (95%CI)^a^**	**P^a^**	**P_inter_^b^**
	**AA + AG**	**GG**			
**AGE**					
≤ 60	1197/1039	194/236	0.69 (0.55–0.86)	0.001	0.733
>60	1222/1302	190/263	0.75 (0.60–0.92)	0.007	
**SEX**					
Male	1324/1302	214/273	0.76 (0.62–0.93)	0.009	0.628
Female	1095/1039	170/226	0.71 (0.56–0.89)	0.003	
**BMI**					
≤ 25	1259/1472	199/309	0.69 (0.56–0.85)	0.001	0.934
>25	1160/869	185/190	0.71 (0.55–0.92)	0.011	
**SMOKING STATUS**					
Yes	807/645	154/131	0.94 (0.71–1.22)	0.621	**0.031**
No	1612/1696	230/368	0.66 (0.54–0.79)	9.67 × 10^−6^	
**DRINKING STATUS**					
Yes	802/573	117/134	0.61 (0.46–0.81)	0.001	0.150
No	1617/1768	267/365	0.79 (0.66–0.94)	0.010	
**HYPERLIPIDEMIA**					
Yes	682/523	147/115	0.96 (0.72–1.28)	0.784	**0.025**
No	1737/1818	237/384	0.66 (0.55–0.79)	6.36 × 10^−6^	
**T2DM**					
Yes	757/614	146/112	1.02 (0.76–1.35)	0.919	**0.010**
No	1662/1727	238/387	0.65 (0.54–0.78)	3.37 × 10^−6^	
**HYPERTENSION**					
Yes	1432/877	227/181	0.74 (0.60-0.92)	0.007	0.909
No	987/1464	157/318	0.73 (0.59-0.90)	0.004	

a Adjusted ORs and P values were obtained from logistic regression analyses after adjusting for age, sex, smoking status, alcohol drinking status, BMI, and histories of hyperlipidemia, T2DM, and hypertension.

b *P–value from the multiplicative likelihood ratio test to assess the potential interaction between SNP rs1136410 and selected variables in CAD risk*.

*Bold values represented statistically significant results, with P_inter_ < 0.05*.

*N, number; OR (95%CI), odds ratio (95% confidence interval); CAD, coronary artery disease; BMI, body mass index*.

**Figure 1:**
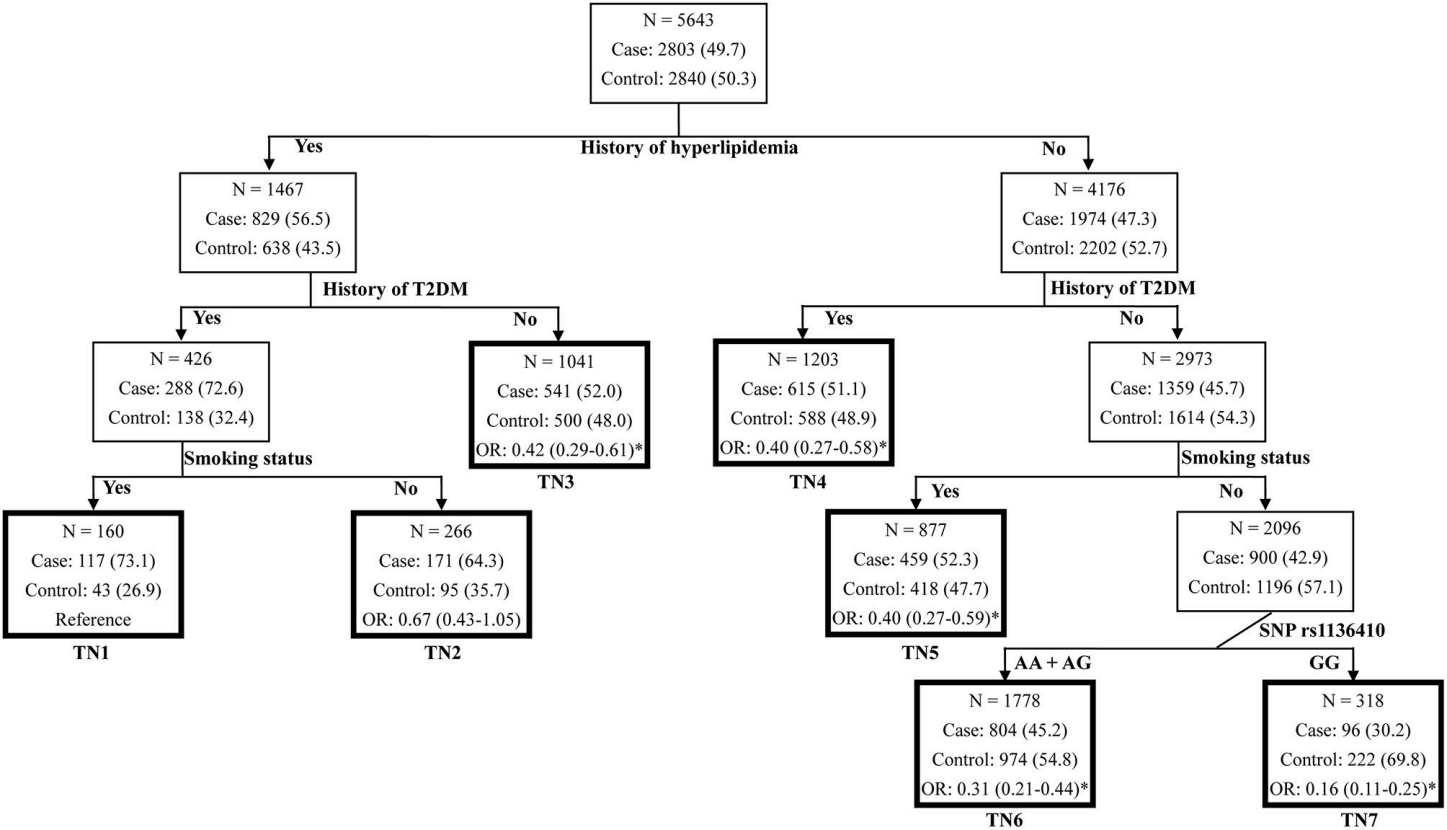
Classification and regression tree for history of hyperlipidemia, history of T2DM, smoking status, and SNP rs1136410 in the merged set. Terminal nodes (TNs) are thick bordered. ORs and 95% CIs were calculated by logistic regression after adjusting for age, sex, smoking status, alcohol drinking status, BMI, and histories of hypertension, hyperlipidemia and T2DM. ^*^P < 0.05.

To further identify the high-order gene-environment interactions in CAD risk, data on SNP rs1136410 and eight traditional CAD risk factors (age >60, male, smoking habit, alcohol drinking habit, BMI >25, hypertension, T2DM, and hyperlipidemia) were included in the MDR analyses. As presented in Table 3, the four-factor model containing “smoking status,” “history of T2DM,” “history of hyperlipidemia,” and “SNP rs1136410” variables were selected as the best interaction model for predicting CAD risk, with the optimal testing accuracy of 0.6773, the maximal CVC of 100/100, and the smallest *P* < 0.0001 in the permutation test. Subsequent CART analyses including these four variables showed that individuals with the variant genotype GG but without smoking habit, T2DM, and hyperlipidemia, were associated with an 84% (OR = 0.16, *P* < 0.001) decreased risk of CAD, compared with the reference group at the greatest risk (Figure 1). Taken together, we suggested the high-order gene-environment interactions among SNP rs1136410, smoking status, history of T2DM, and history of hyperlipidemia in decreasing CAD risk.

**Table 3:** The best models to predict CAD risk by MDR analyses.

**No. of risk factors**	**Best interaction models**	**CVC^a^**	**Testing accuracy (%)^b^**	**Permutation test (*P*-value)^c^**
1	History of hyperlipidemia	74/100	0.6097	0.0102
2	History of hyperlipidemia, history of T2DM	86/100	0.6228	0.0023
3	History of hyperlipidemia, history of T2DM, smoking status	96/100	0.6442	<0.0001
**4**	**History of hyperlipidemia, history of T2DM, smoking status, SNP rs1136410 (AA** + **AG/GG)**	**100/100**	**0.6773**	<**0.0001**
5	History of hyperlipidemia, history of T2DM, smoking status, SNP rs1136410 (AA + AG/GG), history of hypertension	100/100	0.6547	<0.0001
6	History of hyperlipidemia, history of T2DM, smoking status, SNP rs1136410 (AA + AG/GG), history of hypertension, alcohol drinking status	100/100	0.6480	<0.0001
7	History of hyperlipidemia, history of T2DM, smoking status, SNP rs1136410 (AA + AG/GG), history of hypertension, alcohol drinking status, BMI (≤ 25/> 25)	92/100	0.6380	0.0002
8	History of hyperlipidemia, history of T2DM, smoking status, SNP rs1136410 (AA + AG/GG), history of hypertension, alcohol drinking status, BMI (≤ 25/> 25), age (≤ 60/> 60)	91/100	0.6356	0.0003
9	Smoking status, alcohol drinking status, history of T2DM, history of hyperlipidemia, history of hypertension, SNP rs1136410 (AA + AG/GG), BMI (> 25/ <25), age (<60/> 60), sex	95/100	0.6384	0.0001

a *CVC means the number of times that a given combination of factors is identified in each testing set (a total of 100 times)*.

b *Testing accuracy (%) is the percentage of participants for whom a correct prediction is made*.

c *The permutation test was carried out to repeat the MDR analyses 1,000 times and to calculate the CVC and testing accuracy of each n-factor model*.

*Bold values indicate the models that have the maximal CVC and the optimal testing accuracy as well as the most significant P value for permutation test*.

*T2DM, type 2 diabetes mellitus; CVC, cross-validation consistency*.

### Effects of SNP Rs1136410 on CAD severity

**Figure 2:**
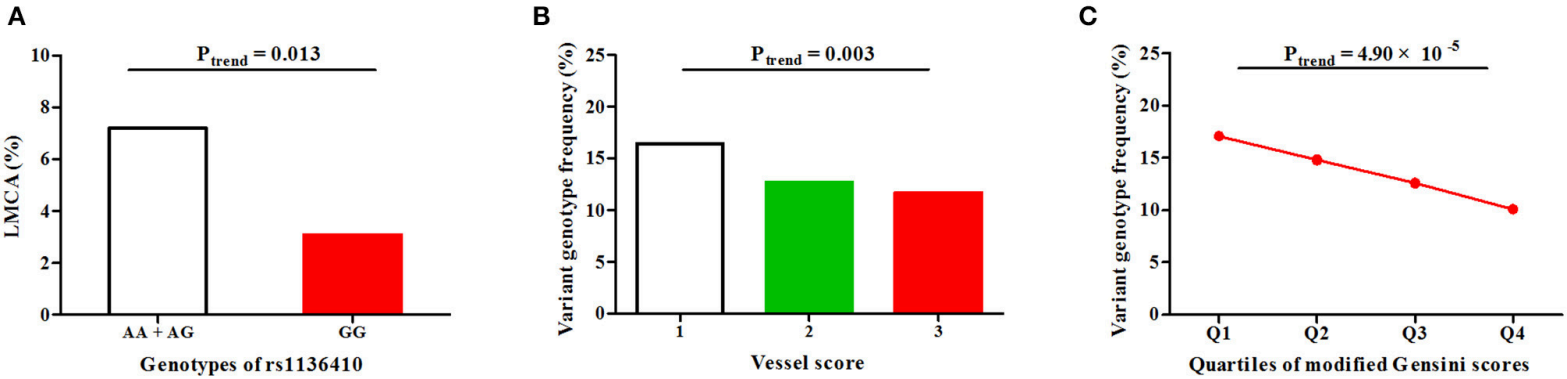
Associations of SNP rs1136410 with CAD severity under a recessive model in the merged set. **(A)** Association of SNP rs1136410 with LMCA lesions; **(B)** Associations between SNP rs1136410 and vessel scores; **(C)** Association of SNP rs1136410 with quartiles of modified Gensini scores. The linear-by-linear association χ^2^ test was used to assess statistical significance.

First, in CAD patients from discovery (*P* = 0.044), replication (*P* = 0.023) and merged sets (*P* = 0.003, Figure 2 and Table S3), the variant genotype GG of SNP rs1136410 was significantly associated with decreased frequencies of LMCA lesions under a recessive model. Then, in replication (*P* = 0.015) and merged sets (*P* = 0.003, Figure 2 and Table S3), the frequencies of the genotype GG showed a decreasing trend from the one-vessel CAD group to the two-vessel CAD group, and ultimately to the three-vessel CAD group. Finally, along with the increasing quartiles (Q1-Q4) of modified Gensini scores, the frequencies of the genotype GG were gradually decreased in patients from all sets (Figure 2 and Table S3).

### Associations of SNP Rs1136410 with lipid levels

In controls from the replication set, linear regression analyses using a recessive model observed a significant association between SNP rs1136410 and TC levels, with a 0.139 mmol/L (*P* = 0.032, Table 4) decrease in TC levels per minor allele. This correlation became more significant in merged controls, with a *P*-value of 0.005 and a β value of −0.132 (Table 4). Furthermore, the genotype GG was consistently associated with increased HDL-c levels in both cases and controls from all sets (Table 4). In merged controls, the variances of TC and HDL-c levels explained by SNP rs1136410 were 0.40 and 0.82%, respectively.

**Table 4:** Associations of SNP rs1136410 with lipid levels in both cases and controls.

**Lipid (mmol/L)**	**Controls**				**CAD**			
	**AA + AG**	**GG**	**β (SE)**	**P^a^**	**AA + AG**	**GG**	**β (SE)**	**P^a^**
**TC**								
Discovery	5.08 ± 0.97	4.96 ± 0.87	−0.124 (0.067)	0.064	5.23 ± 1.06	5.15 ± 0.83	−0.114 (0.082)	0.163
Replication	**5.14** ± **0.98**	**5.01** ± **0.91**	–**0.139 (0.065)**	**0.032**	5.24 ± 0.98	5.15 ± 0.90	−0.082 (0.074)	0.271
Merged	**5.11** ± **0.98**	**4.98** ± **0.89**	–**0.132 (0.047)**	**0.005**	5.24 ± 1.01	5.15 ± 0.86	−0.096 (0.055)	0.081
**HDL-c**								
Discovery	**1.21** ± **0.21**	**1.25** ± **0.23**	**0.032 (0.015)**	**0.031**	**1.00** ± **0.15**	**1.03** ± **0.14**	**0.025 (0.012)**	**0.034**
Replication	**1.20** ± **0.20**	**1.24** ± **0.21**	**0.035 (0.014)**	**0.012**	**1.00** ± **0.16**	**1.03** ± **0.12**	**0.025 (0.012)**	**0.033**
Merged	**1.21** ± **0.20**	**1.25** ± **0.22**	**0.034 (0.010)**	**0.001**	**1.00** ± **0.15**	**1.03** ± **0.13**	**0.026 (0.008)**	**0.002**
**LDL-c**								
Discovery	3.02 ± 0.77	3.02 ± 0.59	0.005 (0.053)	0.917	3.32 ± 0.97	3.26 ± 0.71	−0.084 (0.075)	0.262
Replication	3.03 ± 0.80	3.05 ± 0.77	0.023 (0.055)	0.674	3.24 ± 0.95	3.31 ± 0.69	0.066 (0.071)	0.355
Merged	3.02 ± 0.79	3.04 ± 0.69	0.015 (0.038)	0.702	3.28 ± 0.96	3.28 ± 0.70	−0.002 (0.051)	0.974
**TG**								
Discovery	1.35 ± 0.72	1.34 ± 0.82	−0.014 (0.053)	0.787	1.59 ± 0.84	1.53 ± 0.79	−0.077 (0.066)	0.244
Replication	1.37 ± 0.73	1.36 ± 0.76	−0.003 (0.051)	0.958	1.59 ± 0.84	1.62 ± 0.89	0.029 (0.065)	0.653
Merged	1.36 ± 0.73	1.35 ± 0.79	−0.010 (0.036)	0.774	1.59 ± 0.84	1.58 ± 0.84	−0.017 (0.046)	0.717

a *β (SE) and P values were obtained from multiple linear regression analyses after adjusting for age, sex, smoking status, alcohol drinking status, BMI, and histories of hyperlipidemia, T2DM, and hypertension*.

*Bold values represented statistically significant results, with P < 0.05*.

*TC, total cholesterol; HDL-c, high-density lipoprotein cholesterol; LDL-c, low-density lipoprotein cholesterol; TG, triglyceride*.

### Effects of PARP activities, 8-OHdG levels, and IL-6 levels on CAD risk and severity

**Figure 3:**
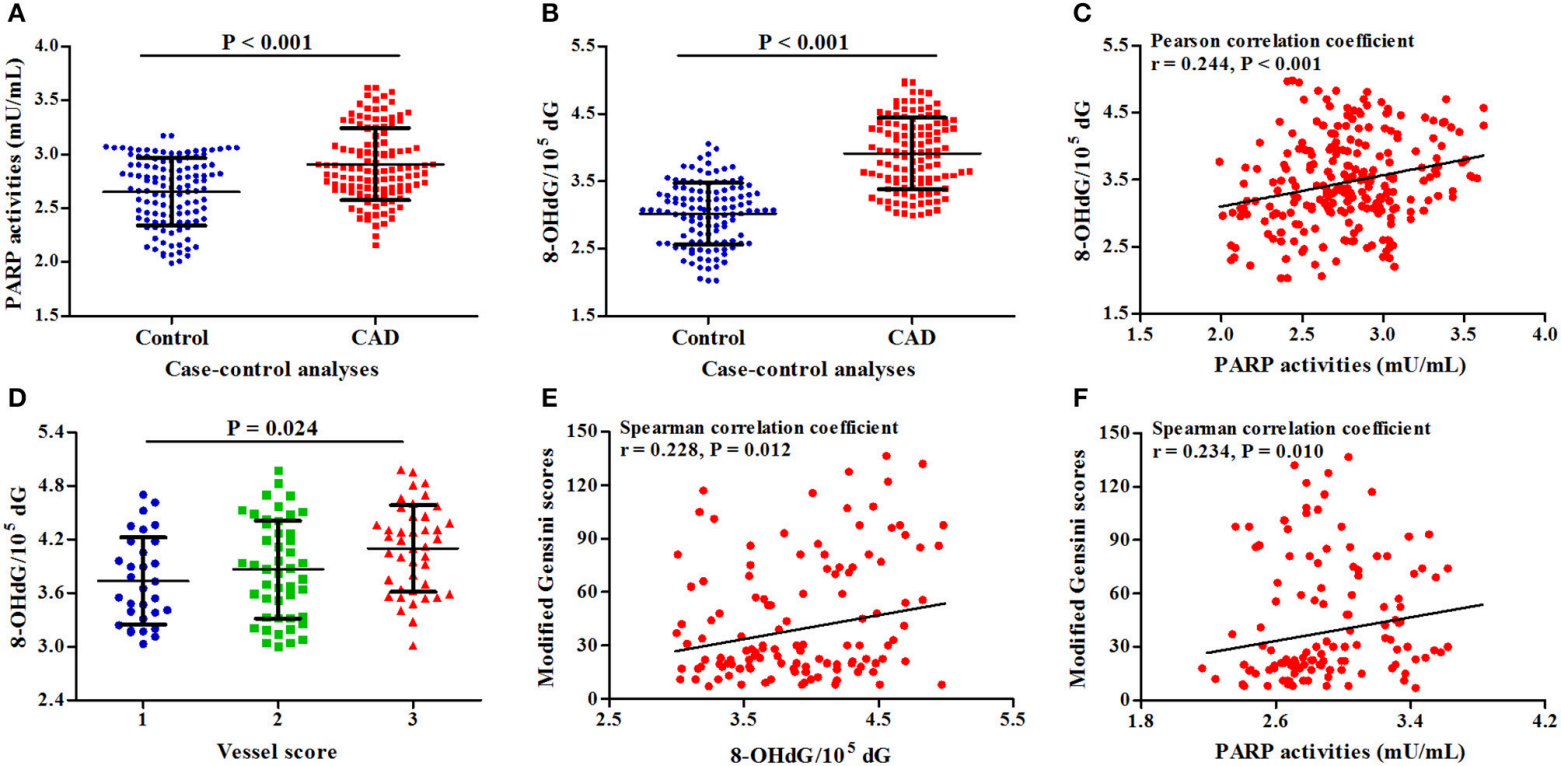
Associations of PARP activities and 8-OHdG levels with CAD risk and severity. **(A)** Difference in PARP activities between cases and controls; **(B)** Difference in 8-OHdG levels between cases and controls; **(C)** Correlation between PARP activities and 8-OHdG levels; **(D)** Association of 8-OHdG levels with vessel scores; **(E)** Correlation between 8-OHdG levels and modified Gensini scores; **(F)** Correlation between 8-OHdG levels and modified Gensini scores.

In two sets of our study, 120 CAD patients and 120 controls were randomly selected to detect PARP activities, 8-OHdG levels, and IL-6 levels in PBMCs (Table S4). As presented in Figures 3A,B, PARP activities and 8-OHdG levels in CAD patients were significantly higher than those in controls (*P* < 0.001). A positive correlation was also found between PARP activities and 8-OHdG levels (*r* = 0.244, *P* < 0.001, Figure 3C). In subtype analyses (Table S5), patients with UAP and MI had increased PARP activities compared with controls; 8-OHdG levels in all CAD subtypes were significantly higher than those in controls.

In CAD patients, along with the increasing levels of 8-OHdG, vessels scores (*P* = 0.024, Figure 3D) and modified Gensini scores (*r* = 0.228, *P* = 0.012, Figure 3E) were gradually increased; there was a positive correlation between PARP activities and modified Gensini scores (*r* = 0.234, *P* = 0.010, Figure 3F).

In PBMCs stimulated by lipopolysaccharide (LPS) (Qin et al., 2016a), IL-6 levels were significantly correlated with CAD risk (*P* < 0.001), 8-OHdG levels (*r* = 0.318, *P* < 0.001), PARP activities (*r* = 0.516, *P* < 0.001), and modified Gensini scores (*r* = 0.226, *P* = 0.013), but not with vessel scores and SNP rs1136410 (Figure S2).

### Associations of SNP Rs1136410 with PARP activities and 8-OHdG levels

**Figure 4:**
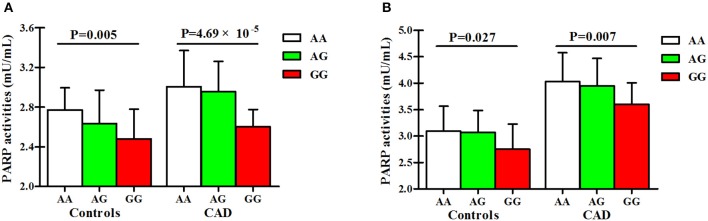
Associations of SNP rs1136410 with PARP activities and 8-OHdG levels. **(A)** Associations of SNP rs1136410 with PARP activities in controls and patients with UAP and MI; **(B)** Associations of SNP rs1136410 with 8-OHdG levels in controls and patients with UAP and MI. *P*-values were obtained from multivariable linear regression models after adjusting for covariates.

As presented in Figure 4 and Table S5, PARP activities and 8-OHdG levels were gradually decreased from the genotype AA to the heterozygous variant genotype AG and ultimately to the homozygous variant genotype GG in both cases and controls.

## Discussion

In SMCs and endothelial cells, hyperactivation of PARP-1 by uncontrolled oxidative DNA damage could trigger a cellular energy crisis and irreversible cell death, which involved the pathogenesis of atherosclerosis (Oumouna-Benachour et al., 2007; Xu et al., 2014). SNP rs1136410 is a non-synonymous mutation, leading to an alanine for valine substitution in the catalytic domain of PARP-1 (Yu et al., 2012). This domain is located in an evolutionarily conserved region, containing a “PARP-signature” motif (Amé et al., 2004; Rouleau et al., 2010; Langelier et al., 2012) which accounts for more than 90% of PARP activities (Aguilar-Quesada et al., 2007). A previous study has suggested a dose-dependent effect of SNP rs1136410 on PARP activities *in vitro* (Wang et al., 2007). In the current study with more than 5,600 participants, we first reported that the variant genotype GG of SNP rs1136410 was associated with decreased CAD risk, reduced CAD severity, lower TC levels, and higher HDL-c levels in a Chinese Han population. Then, MDR and CART analyses further observed the high-order gene-environment interactions among SNP 1136410, smoking status, history of T2DM, and history of hyperlipidemia in decreasing CAD risk. Finally, the genotype-phenotype correlation analyses supported the functional roles of SNP rs1136410 in decreasing PARP activities and 8-OHdG levels. Taken together, we suggest that the minor allele G of SNP rs1136410 may protect against CAD through gene-environment interactions and regulation of PARP activities.

In population studies, Cottet et al. first introduced SNP rs1136410, which was not associated with cellular poly(ADP-ribosyl)ation capacity of PARP-1 in 95 centenarians (Cottet et al., 2000). Then, a larger cohort involving 354 subjects reported an allele dose-dependent impact of SNP rs1136410 on PARP activities (Lockett et al., 2004). Subsequent *in vitro* analyses also suggested functional roles of SNP rs1136410 in maximum velocity and K_M_ values of poly(ADP-ribosyl)ation by PARP-1 (Beneke et al., 2010). In this study, PARP activities were gradually decreased from the genotype AA to the heterozygous variant genotype AG and ultimately to the homozygous variant genotype GG in both cases and controls. Moreover, by integrating the mRNA expression data from ArrayExpress database (https://www.ebi.ac.uk/arrayexpress/experiments/E-MTAB-264/) and the genotyping data from HapMap Project in 160 East Asians (ftp://ftp.ncbi.nlm.nih.gov/hapmap/) (Stranger et al., 2012), we found that carriers not only with the homozygous variant GG, but with the heterozygous variant AG, had significantly lower levels of *PARP-1* mRNA expression than the homozygous wild-type (Table S6). All the evidence above suggests a cumulative effect of SNP rs1136410 on PARP-1 function.

CAD is a continuous process from SAP to UAP and ultimately to MI, which mainly results from the progression of plaque dynamics (Andersson and Vasan, 2014). In functional studies, PARP inhibition or deletion could lead to an increase in collagen content (Oumouna-Benachour et al., 2007), the prevention of dyslipidemia-induced endothelial dysfunction (Hans et al., 2009a), and the reduction in plaque sizes and numbers (Oumouna-Benachour et al., 2007; von Lukowicz et al., 2008; Hans et al., 2009a), suggesting the key roles of PARP activities in plaque progression (Oumouna-Benachour et al., 2007). However, a case-control study from India failed to find a significant association between SNP rs1136410 and MI in patients with T2DM (Narne et al., 2012). Considering the relatively small sample size of Narne et al.'s study (73 MI patients and 121 controls) and the different genetic background between Indians and Chinese (a minor allele frequency of rs1136410: 23.1% in Narne et al.'s controls; 42.7% in our controls), we performed subtype analyses in two sets of our study, and found the protective effects of SNP rs1136410 on MI and UAP. Then, patients with MI and UAP had higher PARP activities than controls. Finally, the variant genotype GG of SNP rs1136410 showed a significantly inverse association with CAD severity, as determined by LMCA lesions, vessel scores, and modified Gensini scores. All these results together indicate that SNP rs1136410 and PARP activities may involve the progression of CAD, and exert greater effects on UAP and MI than on SAP in the Chinese Han population.

In mice, *PARP-1* ablation or treatment of PARP inhibitors could dramatically reduce TC (Hans et al., 2009a; Bai et al., 2011) and LDL-c levels (Hans et al., 2009a,b), elevate HDL-c levels (Hans et al., 2009a), and consequently ameliorate the atherogenic index (Hans et al., 2009a) on either regular or high fat diet. This evidence, combined with the decreased effect of SNP rs1136410 on PARP activities, promoted us to test whether SNP rs1136410 was associated with lipid levels in our population study. We found a constantly strong association between SNP rs1136410 and increased HDL-c levels in both cases and controls, and a modest effect of SNP rs1136410 on decreased TC levels only in controls. Recently, functional studies further showed that PARP inhibition in macrophages enhanced *ABCA1*-induced cholesterol efflux (Shrestha et al., 2016), and increased the detoxification of free cholesterol by down-regulation of *ACAT-1* expression (Hans et al., 2008) (a gene suppressing cholesterol efflux (Hongo et al., 2009). Considering all the evidence above, it is reasonable to speculate that SNP rs1136410 may regulate HDL-c metabolism by reducing PARP activities and subsequently enhancing cholesterol efflux.

In the present study, participants with the variant genotype GG but without smoking habit, T2DM, and hyperlipidemia were associated with an 84% decreased risk of CAD, suggesting the existence of gene-environment interactions. The following evidence may help to interpret this finding. First, as a well-established risk factor for CAD, smoking habit could contribute to oxidative DNA damage and atheroma formation, either directly by invoking peroxidation, or indirectly by consuming endogenous antioxidants (Siasos et al., 2014). Its enhanced effects on oxidative stress may weaken the protective role of SNP rs1136410 in CAD development. Second, *PARP-1* mRNA was overexpressed in T2DM patients (Adaikalakoteswari et al., 2007), and PARP inhibition protected cardiomyocytes from endothelium dysfunction (Choi et al., 2012; Zakaria et al., 2016), glucose stimulation (Choi et al., 2012; Qin et al., 2016b), and apoptosis (Qin et al., 2016b) in mice with T2DM or diabetic cardiomyopathy. So, by reducing PARP activities, SNP rs1136410 may protect against dysglycemia in cardiomyocytes, and further reduce CAD risk. Third, the current study found significant associations of SNP rs1136410 with TC and HDL-c levels, suggesting a potential role of SNP rs1136410 in hyperlipidemia. Taking all the evidence together, we suggest that the protective effect of SNP rs1136410 on CAD risk may greatly enhance in subjects without smoking habit, T2DM, and hyperlipidemia.

Limitations of this study merit further consideration. First, the ELISA kit used for PARP activities only measures newly formed PAR, but not the PAR already presented in the sample, hampering the interpretation of these data. Second, although we analyzed the association between PARP activities and SNP rs1136410, other biomarkers for PARP enzymes, including PARP-1 protein levels and PAR structure, are encouraged to detect. Finally, despite a sufficient statistical power of this study, the Spearman correlation test only showed modest effects of 8-OHdG levels and PARP activities on modified Gensini scores. This finding needs further replication.

In summary, this two-stage case-control study, involving 2803 cases and 2840 controls, shows that the minor allele G of SNP rs1136410 may protect against CAD (risk and severity), reduce TC levels, and increase HDL-c levels through regulation of PARP activities. We also identify the gene-environment interactions among SNP rs1136410, smoking status, history of T2DM, and history of hyperlipidemia in modulating CAD risk. Future studies are warranted to validate these findings and determine the molecular mechanism.

## Author contributions

XW and LM conceived, designed, and performed the study; NC and SG analyzed the data; XW, SZ, and ZL contributed samples/materials/reagents; XW and LM wrote and drafted the manuscript.

### Conflict of interest statement

The authors declare that the research was conducted in the absence of any commercial or financial relationships that could be construed as a potential conflict of interest.
