# Supplementary material for: PARP-1 Variant Rs1136410 Confers Protection against Coronary Artery Disease in a Chinese Han Population: A Two-Stage Case-Control Study Involving 5643 Subjects
Source: Front Physiol. 2017 Nov 14;8:916. doi: 10.3389/fphys.2017.00916 (PMC5694467; doi:10.3389/fphys.2017.00916)
Supplement: Supplementary file 1 [file DataSheet1.DOCX]

**Supplementary Materials**

***PARP-1* variant rs1136410 confers protection against coronary artery disease in a Chinese Han population: a two-stage case-control study involving 5643 subjects**

**Supplementary materials and methods..................................................................2-5**

**Supplementary Figure 1..............................................................................................6**

**Supplementary Figure 2..............................................................................................7**

**Supplementary Tables........................................................................................... 8-13**

Table S1..........................................................................................................................8

Table S2..........................................................................................................................9

Table S3........................................................................................................................10

Table S4........................................................................................................................11

Table S5........................................................................................................................12

Table S6........................................................................................................................13

**Supplementary Materials and Methods**

**Diagnostic Criterion of Different CAD Subtypes**

Stable angina pectoris (SAP) was defined as angiographically confirmed coronary artery disease (CAD), and no change in frequency, duration, or intensity of chest pain in at least 6 weeks at the time of enrollment ([Fraker et al., 2007](#_ENREF_3); [Loh et al., 2014](#_ENREF_4)). Unstable angina pectoris (UAP) was defined as angina of increasing frequency with less exertion and/or at rest or nocturnal and/or severe or prolonged episodes associated with electrocardiographic changes or evidence of ischemia, with negative changes of cardiac biomarkers (creatine kinase MB (CK-MB) and cardiac troponin I (cTnI)) at the time of enrollment ([Fraker et al., 2007](#_ENREF_3); [Loh et al., 2014](#_ENREF_4)). Myocardial infarction (MI) was defined as typical chest pain of ≥ 30 min duration, characteristic electrocardiographic patterns of acute MI, and significant elevation of cardiac biomarkers ([Xu et al., 2014](#_ENREF_12)).

**Assessment of CAD Severity**

In this study, CAD severity was assessed by the presence of left main coronary artery (LMCA) lesions, vessel scores, and modified Gensini scores. LMCA lesions, including the ostium, trunk, and distal diseases, were defined as diameter stenosis of ≥ 50% by angiography or/and a minimal luminal area (MLA) ≤ 7.5 mm^2^ by intravascular ultrasound (IVUS) or/and a fractional flow reserve (FFR) ≤ 0.80 ([Bing et al., 2015](#_ENREF_2); [Ragosta, 2015](#_ENREF_10)). Vessel scores ranged from 1 to 3 according to the number of vessels having ≥ 50% diameter stenosis ([Weintraub et al., 2011](#_ENREF_11)).

In the modified Gensini scoring system ([Montorsi et al., 2006](#_ENREF_7)), angiographic stenosis of each coronary segment was first scored according to the degree of luminal narrowing: 1 for 0-25% stenosis, 2 for 26-50%, 4 for 51-75%, 8 for 76-90%, 16 for 91-99% and 32 for 100%. Then a multiplier was assigned to each segment depending on the functional significance of the area supplied by that segment: 5 for LMCA, 2.5 for proximal left anterior descending coronary artery (LAD) and proximal left circumflex branch, 1.5 for mid-segment of LAD, 0.5 for second diagonal branch and posterolateral branch, and 1 for other branches. Finally, the weighted scores for each segment were added to give modified Gensini scores. As acute coronary occlusion usually occurs in a previous angiographically non-critical lesion, modified Gensini scoring system scores acute total occlusion as a non-critical lesion (0-5 score) instead of true chronic total occlusion (32-172 score).

All the tests were independently evaluated by two experienced cardiologists who were blinded to the clinical and genetic data of each subject. The κ values for inter-observer and intra-observer variability were 0.95 and 0.92 for assessing LMCA lesions, 0.95 and 0.94 for vessel scores, and 0.93, 0.90 for modified Gensini scores, respectively.

**Definition of Clinical Characteristics**

Individuals who smoked ≥ 100 cigarettes in their lifetime were defined as “smokers”, which included ever smokers and current smokers. An ever smoker was a person who had quit smoking at least 1 year prior to interview. Subjects with alcohol consumption at least once a week for ≥ 1 year were defined as “alcohol drinkers”. One drink was defined as 375 ml of beer (13.6 g of ethanol), 118 ml of wine (11.7 g of ethanol), or 30 ml of western or Chinese hard liquor (10.9 g of ethanol). Hypertension was defined as ongoing therapy for hypertension, systolic blood pressure (SBP) of ≥ 140mmHg or diastolic blood pressure (DBP) of ≥ 90mmHg ([Zakopoulos et al., 2013](#_ENREF_14)). Type 2 diabetes mellitus (T2DM) was defined as ongoing therapy for diabetes or fasting plasma glucose (FPG) levels of ≥ 7.0 mmol/L, or plasma glucose levels of ≥ 11.1 mmol/L, or a 2-h plasma glucose level of ≥ 11.1 mmol/L during an oral glucose tolerance test ([Association, 2008](#_ENREF_1)). Hyperlipidemia was defined as hypercholesterolemia (serum TC > 6.2 mmol/L), high levels of LDL-c (> 4.1 mmol/L), low levels of HDL-c (< 1.0 mmol/L), hypertriglyceridemia (serum TG > 2.3 mmol/L) ([National Cholesterol Education Program (NCEP) Expert Panel on Detection, 2002](#_ENREF_8)).

**Measurement of PARP Activities, 8-OHdg Levels, and IL-6 Levels in Peripheral Blood Mononuclear Cells (PBMCs)**

PBMCs were isolated from whole blood samples (7 mL) using the Lymphoprep Solution (Axis-Shield, Oslo, Norway). Protein lysates of PBMCs were obtained by adding lysis buffers (4 mM of NaCl, 1% of Triton X-100, and 200 µM of PMSF). Based on the manufacturer's instructions, PARP activities in 200 ng of total proteins were measured with a HT Colorimetric PARP ELISA kit (Trevigen Inc, Gaithersburg, MD, USA) ([Martin-Oliva et al., 2015](#_ENREF_5)), followed by quantification using a standard curve with the detection range of 0.1-40 mU/mL.

Genomic DNA of PBMCs was extracted using a phenol/chloroform method, followed by digestion using the P1 nuclease (0.2 U/µg), phosphodiesterase I (0.4 U/µg), and alkaline phosphatase (0.04 U/µg) ([Martinet et al., 2002](#_ENREF_6)). The amount of deoxyguanosine (dG) was detected by a Microplate Reader at 254 nm ([Martinet et al., 2002](#_ENREF_6)). The amount of 8-OHdG was determined by a competitive ELISA kit (Trevigen Inc, Gaithersburg, MD, USA), with the detection range of 0.2-80ng/mL. Finally, 8-OHdG levels of PBMC, a well-known biomarker for oxidative DNA damage ([Yang et al., 2014](#_ENREF_13)), were expressed as the ratio of 8-OHdG/10^5^ dG ([Martinet et al., 2002](#_ENREF_6); [Yang et al., 2014](#_ENREF_13)).

To induce IL-6 secretion, PBMCs were first seeded into 24-well culture plates with medium RPMI 1640 (containing 10% fetal calf serum, 100 µg/mL streptomycin, and 100 U/mL penicillin) at 1× 10^6^ cells/mL, and stimulated with 100 ng/mL lipopolysaccharide (LPS) (Sigma-Aldrich, St. Louis, MO, USA) for 24h ([Qin et al., 2016](#_ENREF_9)). Then, IL-6 levels in the supernatants of LPS-treated PBMCs were detected by a competitive ELISA kit (Trevigen Inc, Gaithersburg, MD, USA), with the detection range of 0.156-10 ng/mL.

**Supplementary Figure**


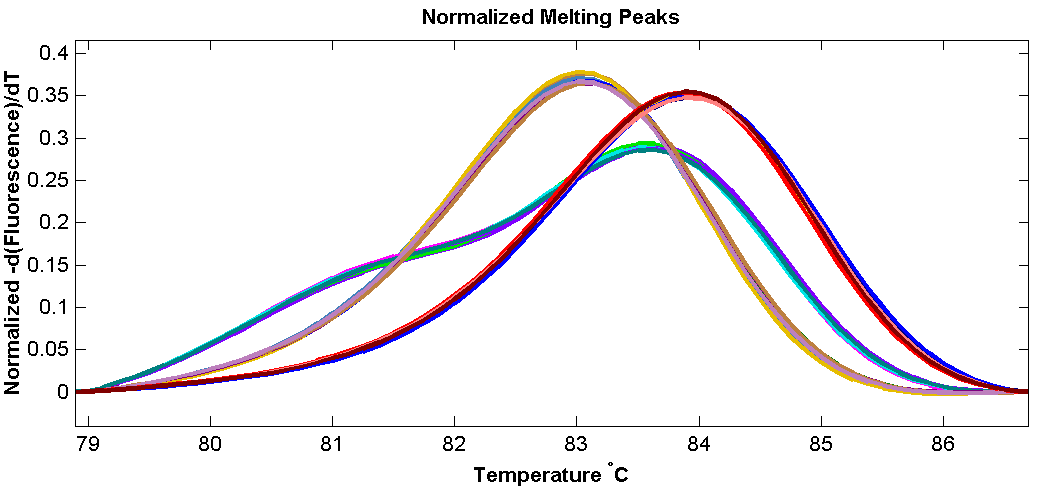

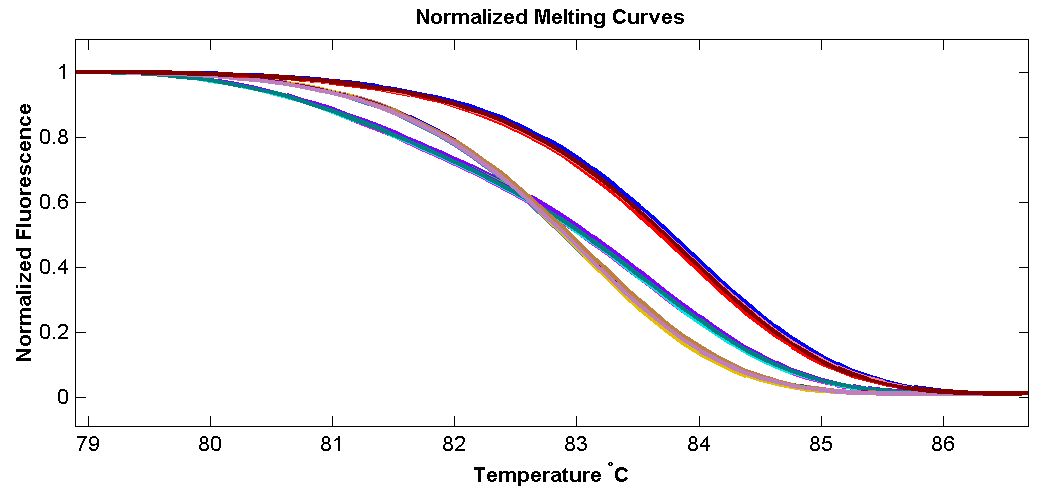


AA

AG

GG

AA

AG

GG

**Figure S1: HRM plots for SNP rs1136410.** The normalized melting peaks are given in the left column, and the normalized melting curves are given in the right column. Arrows indicate the genotypes. Heterozygous samples are identified by a change in melting curve shape, and different homozygotes are distinguished by melting temperature (Tm) shifts.


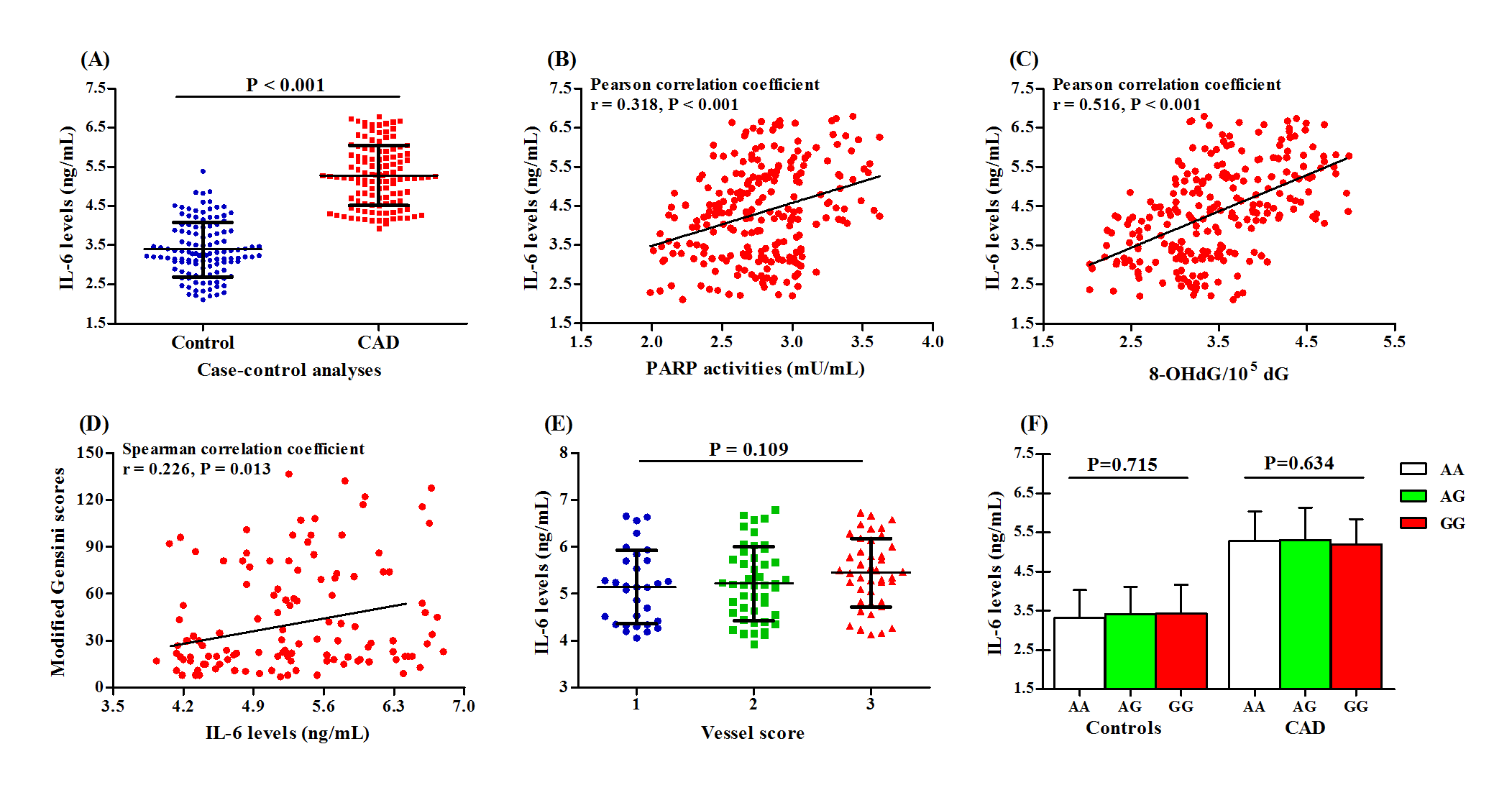


**Figure S2: Associations of IL-6 levels in PBMCs with CAD risk (A), PARP activities (B), 8-OHdG levels (C), modified Gensini scores (D), vessel scores (E), and SNP rs1136410 (F).** P values in Figure S2 (A, E, F) were obtained from multivariable linear regression after adjusting for covariates; P values in Figure S2 (B, C, D) were obtained from Pearson or Spearman correlation test depending on the distribution of the data.

| **Table S1: Clinical characteristics of participants in our study.**  **Supplementary Tables** | | | | | | | | | | | |
| --- | --- | --- | --- | --- | --- | --- | --- | --- | --- | --- | --- |
| Variables | Discovery set (Study 1) | | |  | Replication set (Study 2) | | |  | Merged set (Study 1 + Study 2) | | |
|  | CAD (N = 1266) | Controls (N = 1296) | P ^a^ |  | CAD (N = 1537) | Controls (N = 1544) | P ^a^ |  | CAD (N = 2803) | Controls (N = 2840) | P ^a^ |
| Age, years | 62.9 ± 9.7 | 62.6 ± 10.9 | 0.417 |  | 62.8 ± 9.6 | 62.6 ± 10.7 | 0.592 |  | 62.9 ± 9.7 | 62.6 ± 10.8 | 0.345 |
| Male, n (%) | 711 (56.2) | 712 (54.9) | 0.533 |  | 827 (53.8) | 863 (55.9) | 0.244 |  | 1538 (54.9) | 1575 (55.5) | 0.657 |
| BMI, kg/m^2^ | 25.2 ± 3.9 | 24.1 ± 2.1 | < 0.001 |  | 25.0 ± 3.9 | 24.1 ± 2.1 | < 0.001 |  | 25.1 ± 3.9 | 24.1 ± 2.1 | < 0.001 |
| Smoking, n (%) | 431 (34.0) | 352 (27.2) | < 0.001 |  | 530 (34.5) | 424 (27.5) | < 0.001 |  | 961 (34.3) | 776 (27.3) | < 0.001 |
| Alcohol drinking, n (%) | 409 (32.3) | 344 (26.5) | 0.001 |  | 510 (33.2) | 363 (23.5) | < 0.001 |  | 919 (32.8) | 707 (24.9) | < 0.001 |
| Hypertension, n (%) | 732 (57.8) | 463 (35.7) | < 0.001 |  | 927 (60.3) | 595 (38.5) | < 0.001 |  | 1659 (59.2) | 1058 (37.3) | < 0.001 |
| T2DM, n (%) | 394 (31.1) | 324 (25.0) | 0.001 |  | 509 (33.1) | 402 (26.0) | < 0.001 |  | 903 (32.2) | 726 (25.6) | < 0.001 |
| Hyperlipidemia, n (%) | 381 (30.1) | 288 (22.2) | < 0.001 |  | 448 (29.1) | 350 (22.7) | 0.001 |  | 829 (29.6) | 638 (22.5) | < 0.001 |
| SBP, mmHG | 150.7 ± 34.5 | 133.5 ± 39.1 | < 0.001 |  | 148.4 ± 39.0 | 131.6 ± 20.2 | < 0.001 |  | 149.4 ± 37.0 | 132.5 ± 30.3 | < 0.001 |
| DBP, mmHG | 91.4 ± 17.9 | 82.2 ± 12.1 | < 0.001 |  | 90.9 ± 18.3 | 82.7 ± 12.0 | < 0.001 |  | 91.1 ± 18.1 | 82.5 ± 12.0 | < 0.001 |
| FPG, mmol/L | 5.59 ± 1.58 | 5.26 ± 1.54 | < 0.001 |  | 5.59 ± 1.60 | 5.26 ± 1.54 | < 0.001 |  | 5.59 ± 1.59 | 5.26 ± 1.54 | < 0.001 |
| TC, mmol/L | 5.22 ± 1.02 | 5.06 ± 0.96 | < 0.001 |  | 5.23 ± 0.97 | 5.12 ± 0.97 | 0.002 |  | 5.22 ± 0.99 | 5.09 ± 0.96 | < 0.001 |
| TG, mmol/L | 1.58 ± 0.83 | 1.35 ± 0.74 | < 0.001 |  | 1.59 ± 0.84 | 1.36 ± 0.74 | < 0.001 |  | 1.59 ± 0.84 | 1.36 ± 0.74 | < 0.001 |
| LDL-c, mmol/L | 3.31 ± 0.94 | 3.02 ± 0.74 | < 0.001 |  | 3.25 ± 0.92 | 3.03 ± 0.80 | < 0.001 |  | 3.28 ± 0.93 | 3.03 ± 0.77 | < 0.001 |
| HDL-c, mmol/L | 1.01 ± 0.15 | 1.22 ± 0.21 | < 0.001 |  | 1.00 ± 0.15 | 1.21 ± 0.20 | < 0.001 |  | 1.01 ± 0.15 | 1.22 ± 0.20 | < 0.001 |
| Vessel scores, n (%) |  |  |  |  |  |  |  |  |  |  |  |
| 1 | 436 (34.4) |  |  |  | 546 (35.5) |  |  |  | 982 (35.0) |  |  |
| 2 | 452 (35.7) |  |  |  | 508 (33.1) |  |  |  | 960 (34.2) |  |  |
| 3 | 378 (29.9) |  |  |  | 483 (31.4) |  |  |  | 861 (30.7) |  |  |
| LMCA lesions, n (%) | 91 (7.2) |  |  |  | 96 (6.2) |  |  |  | 187 (6.7) |  |  |
| Modified Gensini score | 30.0 (19.5-70.0) | - |  |  | 31.0 (18.0-75.0) | - |  |  | 30.5 (19.5-72.0) | - |  |
| ^a^ For continuous variables, normally distributed data are expressed as mean ± standard deviation (SD), while skewed data are described as median (interquartile range). For categorical, data are expressed as frequency counts.  Abbreviation: CAD, coronary artery disease; BMI, body mass index; T2DM, type 2 diabetes mellitus; SBP, systolic blood pressure; DBP, diastolic blood pressure; FPG, fasting plasma glucose; TC, total cholesterol; TG, triglyceride; LDL-c, low-density lipoprotein cholesterol; HDL-c, high-density lipoprotein cholesterol; LMCA, left main coronary artery. | | | | | | | | | | | |

| **Table S2: Associations of SNP rs1136410 with CAD subtypes under a recessive model.** | | | | | | | | |
| --- | --- | --- | --- | --- | --- | --- | --- | --- |
| CAD subtypes (case/control, n) | Variant genotype | Frequency (case/control) | Without adjustment | | |  | With adjustment ^a^ | |
|  |  |  | OR (95%CI) | P | P_emp_ ^b^ |  | OR (95%CI) | P |
| SAP (844/2840) | GG | 0.169/0.176 | 0.96 (0.78-1.17) | 0.673 | 0.533 |  | 0.94 (0.76-1.17) | 0.599 |
| UAP (925/2840) | GG | 0.132/0.176 | **0.71 (0.58-0.88)** | **0.002** | **0.002** |  | **0.71 (0.57-0.88)** | **0.002** |
| MI (1034/2840) | GG | 0.115/0.176 | **0.61 (0.49-0.76)** | **6.07 × 10^-6^** | **7.00 × 10^-6^** |  | **0.63 (0.51-0.78)** | **3.59 × 10^-5^** |
| ^a^ Adjusted ORs and P values were obtained from logistic regression analyses after adjusting for age, sex, smoking status, alcohol drinking status, BMI, and histories of hyperlipidemia, T2DM, and hypertension.  ^b^ Emprical P values were obtained from the 100,000-time Monte-Carlo permutation test.  Bold values indicate statistically significant with P < 0.05.  Abbreviation: N, number; CAD, coronary artery disease; SAP, stable angina pectoris; UAP, unstable angina pectoris; MI, myocardial infarction; odds ratio (95% confidence interval). | | | | | | | | |

| **Table S3: Associations of SNP rs1136410 with CAD severity in CAD patients.** | | | | | | | | | | | |
| --- | --- | --- | --- | --- | --- | --- | --- | --- | --- | --- | --- |
| CAD severity | Discovery set (1266 cases) | | |  | Replication set (1537 cases) | | |  | Merged set (2803 cases) | | |
|  | AA + AG | GG | P _trend_ ^a^ |  | AA + AG | GG | P _trend_ ^a^ |  | AA + AG | GG | P _trend_ ^a^ |
| LMCA lesions, n (%) |  |  |  |  |  |  |  |  |  |  |  |
| Yes | 84 (7.8) | 7 (3.7) | **0.044** |  | 91 (6.8) | 5 (2.6) | **0.023** |  | 175 (7.2) | 12 (3.1) | **0.003** |
| No | 993 (92.2) | 182 (96.3) |  |  | 1251 (93.2) | 190 (97.4) |  |  | 2244 (92.8) | 372 (96.9) |  |
| Vessel scores, n (%) |  |  |  |  |  |  |  |  |  |  |  |
| Vessel score-1 | 362 (83.0) | 74 (17.0) | 0.088 |  | 459 (84.1) | 87 (15.9) | **0.015** |  | 821 (83.6) | 161 (16.4) | **0.003** |
| Vessel score-2 | 385 (85.2) | 67 (14.8) |  |  | 453 (89.2) | 55 (10.8) |  |  | 838 (87.3) | 122 (12.7) |  |
| Vessel score-3 | 330 (87.3) | 48 (12.7) |  |  | 430 (89.0) | 53 (11.0) |  |  | 760 (88.3) | 101 (11.7) |  |
| Modified Gensini scores |  |  |  |  |  |  |  |  |  |  |  |
| Quartile-1 | 267 (80.4) | 65 (19.6) | **0.004** |  | 373 (84.8) | 67 (15.2) | **0.005** |  | 640 (82.9) | 132 (17.1) | **4.90 × 10^-5^** |
| Quartile-2 | 269 (85.1) | 47 (14.9) |  |  | 268 (85.4) | 46 (14.6) |  |  | 537 (85.2) | 93 (14.8) |  |
| Quartile-3 | 277 (86.6) | 43 (13.4) |  |  | 342 (88.1) | 46 (11.9) |  |  | 619 (87.4) | 89 (12.6) |  |
| Quartile-4 | 264 (88.6) | 34 (11.4) |  |  | 359 (90.9) | 36 (9.1) |  |  | 623 (89.9) | 70 (10.1) |  |
| Median  (interquartile range) ^b^ | 31.0 (20.0-72.0) | 28.0 (18.0-58.5) | **0.003** |  | 33.0 (19.5-77.0) | 27.0 (18.0-56.0) | **0.005** |  | 32.0 (19.5-74.0) | 27.5 (18.0-56.0) | **3.99 × 10^-5^** |
| ^a^ P _trend_ values were obtained from the linear-by-linear association χ^2^ test.  ^b^ Modified Gensini scores were expressed as median (interquartile range) because of the skewed distributions.  Bold values indicate statistically significant with P _trend_ < 0.05. | | | | | | | | | | | |

| **Table S4: Comparative analyses of clinical and genetic characteristics between the randomly selected subjects and the whole samples.** | | | | | | | |
| --- | --- | --- | --- | --- | --- | --- | --- |
| Variables ^a^ | Control | | |  | CAD | | |
|  | Selected subjects (N = 120) | Whole samples (N = 2840) | P ^b^ |  | Selected subjects (N = 120) | Whole samples (N = 2803) | P ^b^ |
| Age, (year) | 62.9 ± 11.4 | 62.6 ± 10.8 | 0.749 |  | 62.3 ± 10.3 | 62.9 ± 9.7 | 0.499 |
| Male, n (%) | 67 (55.8) | 1575 (55.5) | 0.938 |  | 65 (54.2) | 1538 (54.9) | 0.880 |
| BMI, kg/m^2^ | 24.2 ± 1.9 | 24.1 ± 2.1 | 0.663 |  | 24.8 ± 4.1 | 25.1 ± 3.9 | 0.430 |
| Smoking, n (%) | 27 (22.5) | 776 (27.3) | 0.244 |  | 44 (36.7) | 961 (34.3) | 0.591 |
| Drinking, n (%) | 28 (23.3) | 707 (24.9) | 0.698 |  | 36 (30.0) | 919 (32.8) | 0.524 |
| Hypertension, n (%) | 47 (39.2) | 1058 (37.3) | 0.671 |  | 69 (57.5) | 1659 (59.2) | 0.713 |
| T2DM, n (%) | 27 (22.5) | 726 (25.6) | 0.450 |  | 32 (26.7) | 903 (32.2) | 0.202 |
| Hyperlipidemia, n (%) | 32 (26.7) | 638 (22.5) | 0.281 |  | 30 (25.0) | 829 (29.6) | 0.281 |
| Rs1136410, n (%)‡ |  |  | 0.925 |  |  |  | 0.192 |
| AA | 37 (30.8) | 921 (32.4) |  |  | 33 (27.5) | 978 (34.9) |  |
| AG | 62 (51.7) | 1420 (50.0) |  |  | 66 (55.0) | 1441 (51.4) |  |
| GG | 21 (17.5) | 499 (17.6) |  |  | 21 (17.5) | 384 (13.7) |  |
| Vessel score, n (%) |  |  |  |  |  |  | 0.116 |
| 1 |  |  |  |  | 31 (25.8) | 982 (35.0) |  |
| 2 |  |  |  |  | 47 (39.2) | 960 (34.2) |  |
| 3 |  |  |  |  | 42 (35.0) | 861(30.7) |  |
| LMCA lesions, n (%) |  |  |  |  | 5 (4.2) | 187 (6.7) | 0.278 |
| Modified Gensini score |  |  |  |  | 30.0 (18.0-70.8) | 30.5 (19.5-72.0) | 0.433 |
| ^a^ For continuous variables, normally distributed data were expressed as mean ± SD, while skewed data were described as median (interquartile range). For categorical, data were expressed as frequency counts.  ^b^ The Pearson χ^2^ test and the student t-test (or the Mann-Whitney U test) were used to test for categorical variables and continuous variables, respectively.  Abbreviation: N, number; CAD, coronary artery disease; BMI, body mass index; T2DM, type 2 diabetes mellitus. | | | | | | | |

| **Table S5: Associations of SNP rs1136410 with PARP activities, 8-OHdg levels, and IL-6 levels.** | | | | | | | | | | | | | | |
| --- | --- | --- | --- | --- | --- | --- | --- | --- | --- | --- | --- | --- | --- | --- |
| Markers | Control | |  | CAD | |  | SAP | |  | UAP | |  | MI | |
|  | N | Mean ± SD |  | N | Mean ± SD |  | N | Mean ± SD |  | N | Mean ± SD |  | N | Mean ± SD |
| PARP activities (mU/mL) |  |  |  |  |  |  |  |  |  |  |  |  |  |  |
| Total | 120 | 2.65 ± 0.31 |  | 120 | 2.91 ± 0.33* |  | 32 | 2.69 ± 0.27 |  | 44 | 2.88 ± 0.31* |  | 44 | 3.09 ± 0.30* |
| SNP rs1136410 |  |  |  |  |  |  |  |  |  |  |  |  |  |  |
| AA | 37 | 2.77 ± 0.22 |  | 33 | 3.01 ± 0.36 |  | 7 | 2.65 ± 0.29 |  | 13 | 2.97 ± 0.29 |  | 13 | 3.24 ± 0.30 |
| AG | 62 | 2.64 ± 0.33 |  | 66 | 2.96 ± 0.30 |  | 16 | 2.77 ± 0.27 |  | 23 | 2.93 ± 0.32 |  | 27 | 3.09 ± 0.26 |
| AA + AG | 99 | 2.69 ± 0.30 |  | 99 | 2.97 ± 0.32 |  | 23 | 2.73 ± 0.28 |  | 36 | 2.94 ± 0.30 |  | 40 | 3.13 ± 0.28 |
| GG | 21 | 2.48 ± 0.30** |  | 21 | 2.60 ± 0.18** |  | 9 | 2.59 ± 0.25 |  | 8 | 2.58 ± 0.12** |  | 4 | 2.67 ± 0.06** |
| 8-OHdG/10^5^ dG |  |  |  |  |  |  |  |  |  |  |  |  |  |  |
| Total | 120 | 3.02 ± 0.46 |  | 120 | 3.91 ± 0.53* |  | 32 | 3.85 ± 0.47* |  | 44 | 3.87 ± 0.55* |  | 44 | 3.99 ± 0.55* |
| SNP rs1136410 |  |  |  |  |  |  |  |  |  |  |  |  |  |  |
| AA | 37 | 3.09 ± 0.47 |  | 33 | 4.03 ± 0.55 |  | 7 | 4.02 ± 0.48 |  | 13 | 3.95 ± 0.61 |  | 13 | 4.12 ± 0.55 |
| AG | 62 | 3.07 ± 0.41 |  | 66 | 3.95 ± 0.52 |  | 16 | 3.84 ± 0.55 |  | 23 | 3.93 ± 0.52 |  | 27 | 4.03 ± 0.51 |
| AA + AG | 99 | 3.08 ± 0.44 |  | 99 | 3.98 ± 0.53 |  | 23 | 3.90 ± 0.52 |  | 36 | 3.94 ± 0.54 |  | 40 | 4.06 ± 0.52 |
| GG | 21 | 2.75 ± 0.48** |  | 21 | 3.60 ± 0.40** |  | 9 | 3.75 ± 0.29 |  | 8 | 3.56 ± 0.49** |  | 4 | 3.36 ± 0.40** |
| IL-6 levels (ng/mL) |  |  |  |  |  |  |  |  |  |  |  |  |  |  |
| Total | 120 | 3.39 ± 0.70 |  | 120 | 5.25 ± 0.82* |  | 32 | 5.20 ± 0.76* |  | 44 | 5.39 ± 0.75* |  | 44 | 3.99 ± 0.55* |
| SNP rs1136410 |  |  |  |  |  |  |  |  |  |  |  |  |  |  |
| AA | 37 | 3.33 ± 0.70 |  | 33 | 5.28 ± 0.76 |  | 7 | 4.85 ± 0.82 |  | 13 | 5.21 ± 0.68 |  | 13 | 5.59 ± 0.72 |
| AG | 62 | 3.42 ± 0.69 |  | 66 | 5.31 ± 0.82 |  | 16 | 5.38 ± 0.89 |  | 23 | 5.22 ± 0.84 |  | 27 | 5.34 ± 0.79 |
| AA + AG | 99 | 3.38 ± 0.69 |  | 99 | 5.30 ± 0.80 |  | 23 | 5.22 ± 0.88 |  | 36 | 5.22 ± 0.77 |  | 40 | 5.42 ± 0.77 |
| GG | 21 | 3.43 ± 0.73 |  | 21 | 5.20 ± 0.64 |  | 9 | 5.31 ± 0.69 |  | 8 | 5.12 ± 0.73 |  | 4 | 5.10 ± 0.40 |
| * P < 0.05, in the comparisons between CAD patients and healthy controls. ** P < 0.05, in the comparisons between different genotypes of SNP rs1136410.  Abbreviation: CAD, coronary artery disease; N, number; SD, standard deviation. | | | | | | | | | | | | | | |

| **Table S6: Association of SNP rs1136410 with PARP-1 mRNA expression in 160 east Asians from HapMap Project.** | | | | |
| --- | --- | --- | --- | --- |
| rs1136410 ^a^ | PARP-1 mRNA expression ^b^ | | P ^c^ | P_trend_ ^c^ |
|  | N | Mean ± SD |  |  |
| AA | 44 | 14.33 ± 0.24 | - | 0.033 |
| AG | 82 | 14.22 ± 0.25 | 0.026 |  |
| GG | 34 | 14.19 ± 0.26 | 0.019 |  |
| ^a^ The genotype data were extracted from the HapMap Phase 3.3, CHB and JPT datasets, involving 160 east Asian (79 Chinese subjects and 81 Japanese subjects).  ^b^ The mRNA expression data were acquired from ArrayExpress E-MTAB-264 dataset, which contained the RNA-seq data in the same 160 east Asians.  ^c^ P and P_trend_ values obtained from one-way ANOVA analysis, followed by Fishers’s least significant difference (LSD) test for pairwise comparisons. | | | | |

**Supplementary References**

Association, A.D. (2008). Standards of medical care in diabetes--2008. *Diabetes Care* 31 Suppl 1**,** S12-54. doi: 10.2337/dc08-S012.

Bing, R., Yong, A.S., and Lowe, H.C. (2015). Percutaneous Transcatheter Assessment of the Left Main Coronary Artery: Current Status and Future Directions. *JACC Cardiovasc Interv* 8(12)**,** 1529-1539. doi: 10.1016/j.jcin.2015.07.017.

Fraker, T.D., Jr., Fihn, S.D., Gibbons, R.J., Abrams, J., Chatterjee, K., Daley, J., et al. (2007). 2007 chronic angina focused update of the ACC/AHA 2002 guidelines for the management of patients with chronic stable angina: a report of the American College of Cardiology/American Heart Association Task Force on Practice Guidelines Writing Group to develop the focused update of the 2002 guidelines for the management of patients with chronic stable angina. *J Am Coll Cardiol* 50(23)**,** 2264-2274. doi: 10.1016/j.jacc.2007.08.002.

Loh, J.P., Pendyala, L.K., Torguson, R., Chen, F., Satler, L.F., Pichard, A.A., et al. (2014). Incidence and correlates of major bleeding after percutaneous coronary intervention across different clinical presentations. *Am Heart J* 168(3)**,** 248-255. doi: 10.1016/j.ahj.2014.05.018.

Martin-Oliva, D., Martin-Guerrero, S.M., Matia-Gonzalez, A.M., Ferrer-Martin, R.M., Martin-Estebane, M., Carrasco, M.C., et al. (2015). DNA damage, poly(ADP-Ribose) polymerase activation, and phosphorylated histone H2AX expression during postnatal retina development in C57BL/6 mouse. *Invest Ophthalmol Vis Sci* 56(2)**,** 1301-1309. doi: 10.1167/iovs.14-15828.

Martinet, W., Knaapen, M.W., De Meyer, G.R., Herman, A.G., and Kockx, M.M. (2002). Elevated levels of oxidative DNA damage and DNA repair enzymes in human atherosclerotic plaques. *Circulation* 106(8)**,** 927-932.

Montorsi, P., Ravagnani, P.M., Galli, S., Rotatori, F., Veglia, F., Briganti, A., et al. (2006). Association between erectile dysfunction and coronary artery disease. Role of coronary clinical presentation and extent of coronary vessels involvement: the COBRA trial. *Eur Heart J* 27(22)**,** 2632-2639. doi: 10.1093/eurheartj/ehl142.

National Cholesterol Education Program (NCEP) Expert Panel on Detection, E., and Treatment of High Blood Cholesterol in Adults (Adult Treatment Panel III) (2002). Third Report of the National Cholesterol Education Program (NCEP) Expert Panel on Detection, Evaluation, and Treatment of High Blood Cholesterol in Adults (Adult Treatment Panel III) final report. *Circulation* 106(25)**,** 3143-3421.

Qin, J., Li, L., Zhang, D., Yu, H., Tan, H., Zhang, J., et al. (2016). Analysis of receptor tyrosine kinase genetics identifies two novel risk loci in GAS6 and PROS1 in Behcet's disease. *Sci Rep* 6**,** 26662. doi: 10.1038/srep26662.

Ragosta, M. (2015). Left main coronary artery disease: importance, diagnosis, assessment, and management. *Curr Probl Cardiol* 40(3)**,** 93-126. doi: 10.1016/j.cpcardiol.2014.11.003.

Weintraub, W.S., Karlsberg, R.P., Tcheng, J.E., Boris, J.R., Buxton, A.E., Dove, J.T., et al. (2011). ACCF/AHA 2011 key data elements and definitions of a base cardiovascular vocabulary for electronic health records: a report of the American College of Cardiology Foundation/American Heart Association Task Force on Clinical Data Standards. *J Am Coll Cardiol* 58(2)**,** 202-222. doi: 10.1016/j.jacc.2011.05.001.

Xu, C., Yang, Q., Xiong, H., Wang, L., Cai, J., Wang, F., et al. (2014). Candidate pathway-based genome-wide association studies identify novel associations of genomic variants in the complement system associated with coronary artery disease. *Circ Cardiovasc Genet* 7(6)**,** 887-894. doi: 10.1161/circgenetics.114.000738.

Yang, S., Wu, H., Zhao, J., Wu, X., Zhao, J., Ning, Q., et al. (2014). Feasibility of 8-OHdG formation and hOGG1 induction in PBMCs for assessing oxidative DNA damage in the lung of COPD patients. *Respirology* 19(8)**,** 1183-1190. doi: 10.1111/resp.12378.

Zakopoulos, N., Manios, E., and Moulopoulos, S. (2013). Guidelines for the management of hypertension and target organ damage. *J Hypertens* 31(12)**,** 2463-2464. doi: 10.1097/hjh.0000000000000003.
